# The risk prediction model for acute urine retention after perineal prostate biopsy based on the LASSO approach and Boruta feature selection

**DOI:** 10.3389/fonc.2025.1626529

**Published:** 2025-09-11

**Authors:** Cheng Shen, Gen Chen, Zhan Chen, Junjie You, Bing Zheng

**Affiliations:** ^1^ Department of Urology, Affiliated Hospital 2 of Nantong University, Nantong, Jiangsu, China; ^2^ Jiangsu Nantong Urological Clinical Medical Center, Nantong, Jiangsu, China; ^3^ Institute of Urological Diseases, Nantong University, Nantong, Jiangsu, China; ^4^ Department of Urology, Nantong Second People’s Hospital, Nantong, Jiangsu, China; ^5^ Department of Urology, Rudong Hospital, Xinglin College of Nantong University, Nantong, Jiangsu, China

**Keywords:** acute urinary retention, prostate biopsy, machine learning, predictive model, Boruta feature selection

## Abstract

**Objective:**

One known side effect of transperineal (TP) prostate biopsies is acute urine retention (AUR). We aimed to create and evaluate a predictive model for the post-paracentesis risk of acquiring AUR.

**Methods:**

This study included 599 patients undergoing prostate biopsies (April 2020-July 2023) at the Second Affiliated Hospital of Nantong University, selected based on abnormal digital rectal examination and/or PSA (prostate-specificantigen) > 4 ng/mL. Acute urinary retention (AUR) was defined as the inability to void within 72 hours post-biopsy, requiring catheterization. Patients were randomly divided into training (419 cases) and test (180 cases) sets. Univariate logistic analysis and feature selection Boruta and LASSO (Least absolute shrinkage and selection operator) identified predictors, followed by multivariate logistic regression to develop a predictive nomogram for AUR. Internal validation used the test set, with model performance assessed via the c-index, ROC (Receiver Operating Characteristic) curve, calibration plot, and decision curve analysis. The nomogram demonstrated strong discrimination, calibration, and clinical utility for AUR risk prediction.

**Results:**

In 86 patients (14.3%), AUR happened. An examination of multivariate logistic regression revealed six distinct risk variables for AUR. Based on these independent risk factors, a nomogram was constructed. The training and validation groups’ c-indices showed the model’s high accuracy and stability. The calibration curve demonstrates that the corrective effect of the training and verification groups is perfect, and the area under the receiver operating characteristic curve indicates great identification capacity. DCA (Decision Curve Analysis) curves, or decision curve analysis, demonstrated the model’s significant net therapeutic effect.

**Discussion:**

The nomogram model created in this work can offer a personalized and intuitive analysis of the risk of AUR and has intense discrimination and accuracy. It can help create efficient preventative measures and identify high-risk populations.

## Introduction

In 2022, prostate cancer accounted for around 27% of all new cancer diagnoses in the United States and was the most common cancer diagnosis among men (1). Research has focused on prostate biopsies as the gold standard for diagnosing prostate cancer in recent years. There are two methods for prostate biopsies: transrectal (TR) and transperineal (TP) (2). TR has historically carried out prostate biopsies. The burden that postoperative sepsis places on patient health and adds to the expenditures to the healthcare system, however, is the primary critique of this strategy (3–6). As a result of the declining percentage of TP biopsy in sepsis cases, there has been a nationwide movement to offer TP biopsy. AUR, or acute urinary retention, is another danger associated with prostate biopsies that may be higher than other TR techniques (7–10). AUR frequently necessitates catheterization and additional hospitalization (11, 12). While TP biopsy may lessen infection, it does not entirely prevent infection. According to studies referenced by NICE, urinary retention rates range from 1.6 to 11.4% (12).

Data evaluating possible risk factors for AUR after prostate biopsy in TP are currently hard to come by (13–15). Because medical big data is so common, machine learning (ML), the most significant artificial intelligence implementation technique, has been applied extensively to data-driven risk prediction (16, 17). Given the high dimensionality of the data, we chose to use LASSO first for preliminary screening to reduce Boruta ‘s computational burden and improve the efficiency of the overall analysis. Therefore, after screening out risk factors using LASSO regression. We further screened variables using Boruta ‘s feature screening method. We then iteratively processed random fluctuations in forest importance scores and factor interactions to screen for significant urinary retention predictors. In addition, this approach is commonly used for feature selection in diabetes mellitus (DM) studies (18, 19). In addition, Meng Zirui et al. constructed predictive models for severe novel coronavirus pneumonia using variant analysis and least absolute contraction and selection operator (LASSO) modeling along with Boruta ‘s algorithm (20). Ding Xuexuan et al. identified the core genes of asthma using five machine learning algorithms: LASSO, SVM-RFE, Boruta, XGBoost, and RF (21).

In view of the high clinical incidence of acute urinary retention (AUR) after transperineal prostate puncture (TP) and the lack of a precise predictive model for this complication, the aim of this study was to construct and validate a nomogram model that can be used to rapidly assess the risk of AUR preoperatively by integrating clinical routine indicators (such as BMI, prostate volume, etc.) using LASSO and Boruta feature selection algorithms to provide a quantitative tool for clinical decision-making in order to reduce the postoperative catheter indwelling rate and length of hospital stay.

## Patients and methods

### Study design and participants

We conducted a retrospective analysis of a cohort of patients who underwent prostate biopsy in the day ward of the Department of Urology, the First People’s Hospital of Nantong, from April 2020 to July 2023. Abnormal digital rectal examination, high PSA (> 4.0 ng/mL), or positive prostate multiparametric MRI (prostate imaging report and data system ≥ 3) were the inclusion criteria. The following conditions precluded study participation: hypersensitivity to ciprofloxacin, prostate-related surgery within the previous three months, urinary tract infection during biopsy or therapy, and denial of informed written consent. The study excluded patients with a history of urine retention and those with urinary retention during biopsy. Data parameters included patient demographics, PSA readings, the International Prostate Symptom Score (IPSS), prostate volume, post-void residual (PVR) volume before biopsy, comorbidities, blood and urine routines, post-void residual (PVR) volume, and histopathological findings.

### Ethics and informed consent

The research adhered to the principles outlined in the Declaration of Helsinki. All subjects gave informed consent, which was approved by the First People’s Hospital Center of Nantong’s Ethics Committee (ethical approval number 2022KT100). Because this study was a retrospective cross-sectional study, informed consent was not required and all subjects included in the study had signed an informed consent form authorizing the use of the information for future scientific research. Retrospective data analysis followed the ethical guidelines applicable at the time of source data collection (Declaration of Helsinki 2013 revision).

### Data collection and variable definition

Demographic information was obtained from electronic medical records, along with PSA values, the International Prostate Symptom Score (IPSS), prostate volume, post-void residual (PVR) volume before biopsy, comorbidities, blood and urine routines, routine inflammatory parameters, and post-void residual (PVR) volume. Before being included in the study, a second reviewer verified the chart review data’s accuracy. We used multiple imputation to handle missing values, the number of imputations was 5, and variables with < 5% missing proportion were finally retained; abnormal values (identified by Z-score method, | Z | > 3) were verified, corrected after confirming abnormal values due to measurement error, and the rest retained original data to avoid information loss; normality tests were performed for continuous variables (e.g., prostate volume, IPSS score), and logarithmic transformation was used for non-normally distributed variables (e.g., residual urine volume) to ensure the rationality of model input data. AUR was defined as the inability to void within 72 hours following biopsy, necessitating the implantation of a urinary catheter (22).

### Histopathologic evaluation

All biopsies were analyzed by 2 urogenital pathologists (> 10 years’ experience). The location, proportion of cancer tissue per core, and Gleason score (GS), based on the 2005 consensus of the International Society of Uropathology (23), were recorded for each prostate cancer-positive biopsy core.

### PI-RADS score

Before having a prostate biopsy, all patients had 3.0 T mpMRIs (no endorectal coils). T1-weighted imaging (T1WI), T2-weighted imaging (T2WI), diffusion-weighted imaging (DWI), and dynamic contrast-enhanced imaging (DCE) are among the scanning techniques used for mpMRI. After DWI data were collected at b-values of 0 and 1500 s/mm 2, ADC maps were produced. Two genitourinary radiologists with at least three years of prostate MRI expertise evaluated the mpMRI, and the PI-RADSv2.1 score was used to record the results. For CSPCa, it is doubtful to originate in PI-RADS 1 (doubtful to occur in CSPCa), PI-RADS 2 (unlikely to happen in CSPCa), and PI-RADS 3 (suspicious for CSPCa); nevertheless, for PI-RADS 4 (high) and PI-RADS 5 (very high) (24, 25).

### Statistical analysis

The continuous data were evaluated using the Student’s t-test or the Mann-Whitney U test, and the results were reported as mean ± standard deviation (SD) or median and interquartile range. Alternatively, categorical data reported as numbers (%) was evaluated using Fisher’s exact or Chi-square tests. We used LASSO regression to reduce the dimension of high-dimensional data and identify the best predictive characteristics and variables of AUR after performing univariate logistic regression analysis of the training group to first screen the factors and determine the risk factors of AUR (26). In addition, we employed Boruta’s technique for feature selection, which involved 100 random forest iterations and the creation of logistic regression prediction models. Nomograms are validated by measuring their calibration (calibration graph) and discriminant capacity (C-statistic). C-statistic values above 0.75 are generally indicative of comparatively excellent discriminant ability. Lastly, we used decision curve analysis (DCA) to assess how applicable nomograms are in clinical practice. In every analysis, a p-value of less than 0.05 was deemed statistically significant.

## Results

### Clinical features

**Figure 1:**
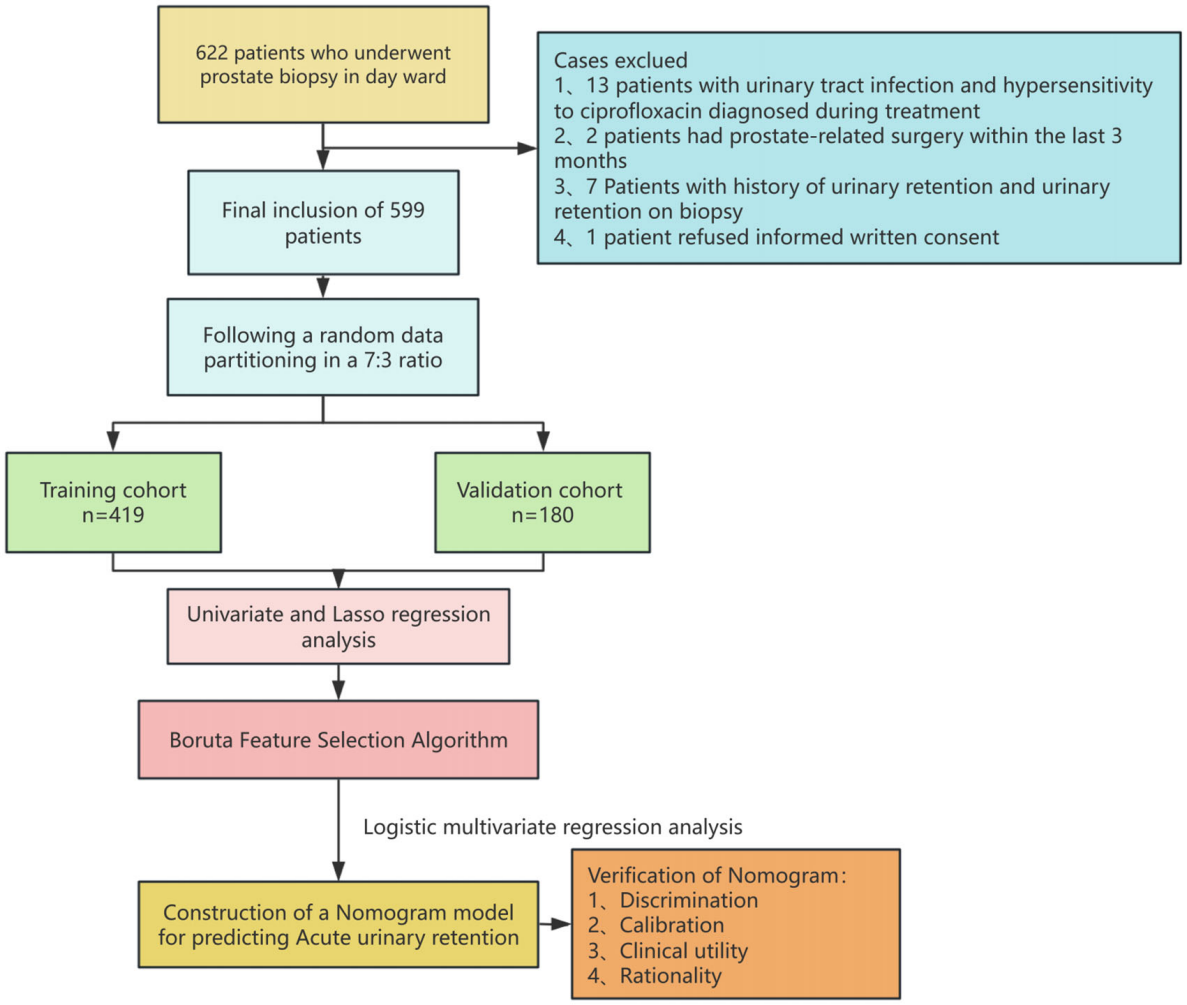
Research pathway diagram.

Between January 1, 2020, and December 31, 2023, 599 patients who underwent prostate biopsies in Urology Department Day Ward at the Second Affiliated Hospital of Nantong University provided data for our analysis. Of these, 513 cases (85.64%) did not experience postoperative acute urine retention, while 86 (14.36%) experienced it. **Figure 1** is a flow chart of the case selection and study process. The patients’ demographic features are detailed in **Table 1**.

**Table 1:** Baseline characteristics of patients.

Characteristics	Total (N=599)	No AUR (N=513)	AUR (N=86)	P	Statistic
Age(year)	72.00[67.00;77.00]	72.00 [67.00;77.00]	73.00 [68.00;76.00]	0.837	21753
BMI(kg/m^2)	23.63[21.33;24.80]	23.41 [21.19;24.74]	24.59 [23.54;26.35]	<0.001	13189.5
Serum PSA(ng/ml)	13.34 [7.56;23.73]	13.34 [7.56;23.73]	16.50 [11.24;22.69]	0.018	18536
Prostate volume(ml)	40.77[28.66;57.42]	37.62 [27.95;49.76]	98.17 [59.71;125.70]	<0.001	6894
Urine leukocyte	17.00 [9.00;24.00]	17.00 [9.00;24.00]	17.00 [11.00;22.00]	0.552	22942
Blood White cell	6.90 [5.50;7.80]]	6.90 [5.50;7.80]	7.15 [5.82;7.77]	0.979	22020.5
History of Electrocision				0.161	1.966
NO	494 (82.47%)	418 (81.48%)	76 (88.37%)		
Yes	105 (17.53%)	95 (18.52%)	10 (11.63%)		
Diabetes				<0.001	54.496
NO	405 (67.61%)	377 (73.49%)	28 (32.56%)		
Yes	194 (32.39%)	136 (26.51%)	58 (67.44%)		
Constipation				<0.001	53.505
NO	398 (66.44%)	371 (72.32%)	27 (31.40%)		
Yes	201 (33.56%)	142 (27.68%)	59 (68.60%)		
Before biopsy PVR(ml)	63.00 [42.00;89.00]	56.00 [42.00;78.00]	114.00 [88.00;156.00]	<0.001	7272
IPSS	12.00 [8.00;19.00]	12.00 [7.00;17.00]	22.00 [12.00;28.00]	<0.001	11758
Repeated biopsies			6	0.006	7.536
NO	519 (86.64%)	453 (88.30%)	66 (76.74%)		
Yes	80 (13.36%)	60 (11.70%)	20 (23.26%)		
PI-RADS Score				0.623	0.946
3	184 (30.72%)	159 (30.99%)	25 (29.07%)		
4	223 (37.23%)	187 (36.45%)	36 (41.86%)		
5	192 (32.05%)	167 (32.55%)	25 (29.07%)		
Target Location				<0.001	17.394
NO	316 (52.75%)	289 (56.34%)	27 (31.40%)		
Yes	283 (47.25%)	224 (43.66%)	59 (68.60%)		
Number of needles				0.837	0.042
12	76 (12.69%)	64 (12.48%)	12 (13.95%)		
16	523 (87.31%)	449 (87.52%)	74 (86.05%)		
Prostate Ca				0.901	0.015
NO	216 (36.06%)	186 (36.26%)	30 (34.88%)		
Yes	383 (63.94%)	327 (63.74%)	56 (65.12%)		
Histopathologic inflammation				<0.001	24.85
NO	413 (68.95%)	374 (72.90%)	39 (45.35%)		
Yes	186 (31.05%)	139 (27.10%)	47 (54.65%)		

PSA, prostate-specific antigen; AUR, Acute urinary retention; IPSS, International Prostate Symptom Score.

### Risk factors associated with AUR

**Figure 2:**
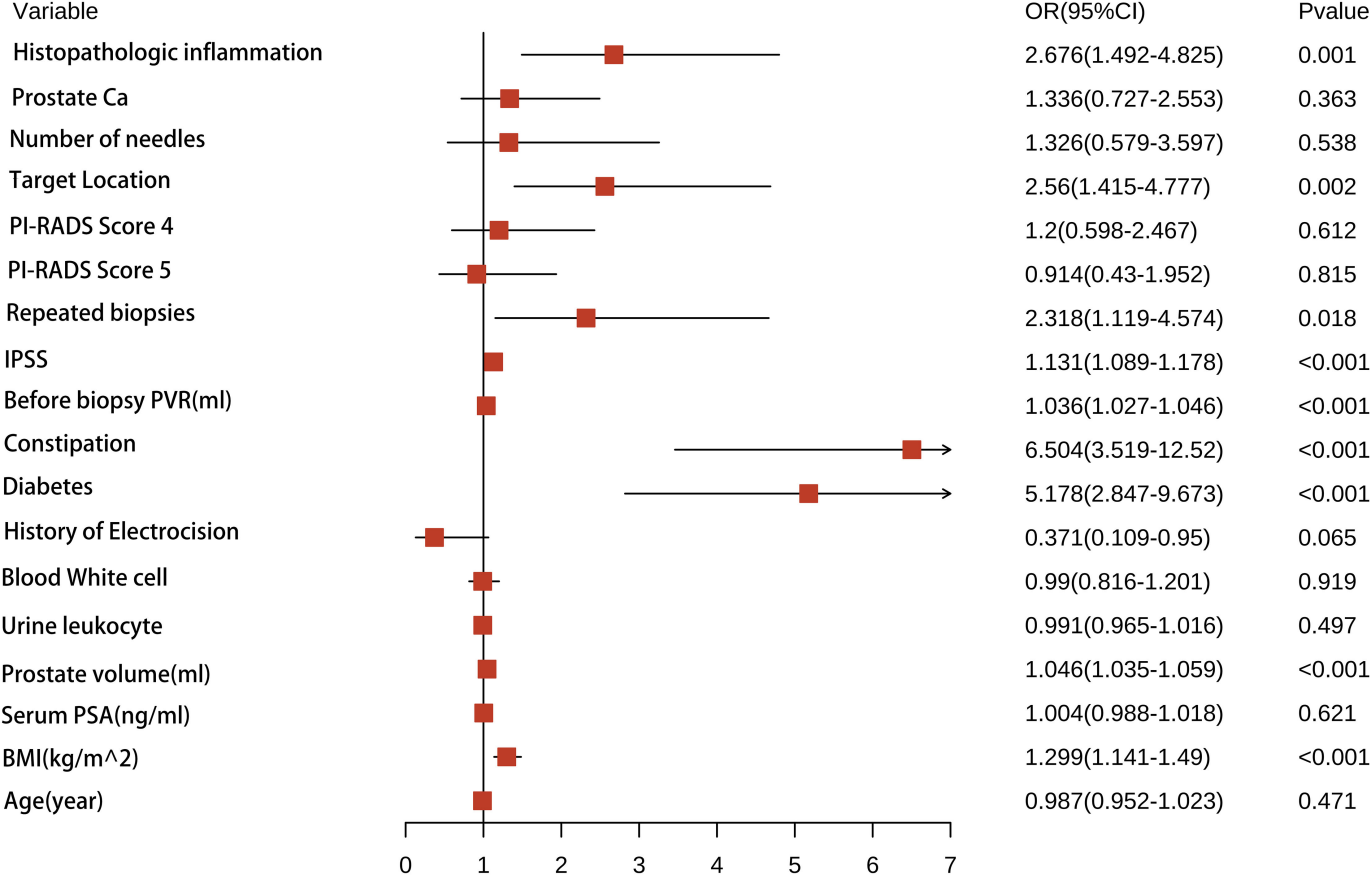
A forest plot illustrating the all of characteristics identified by univariate logistic regression analyses.

**Figure 3:**
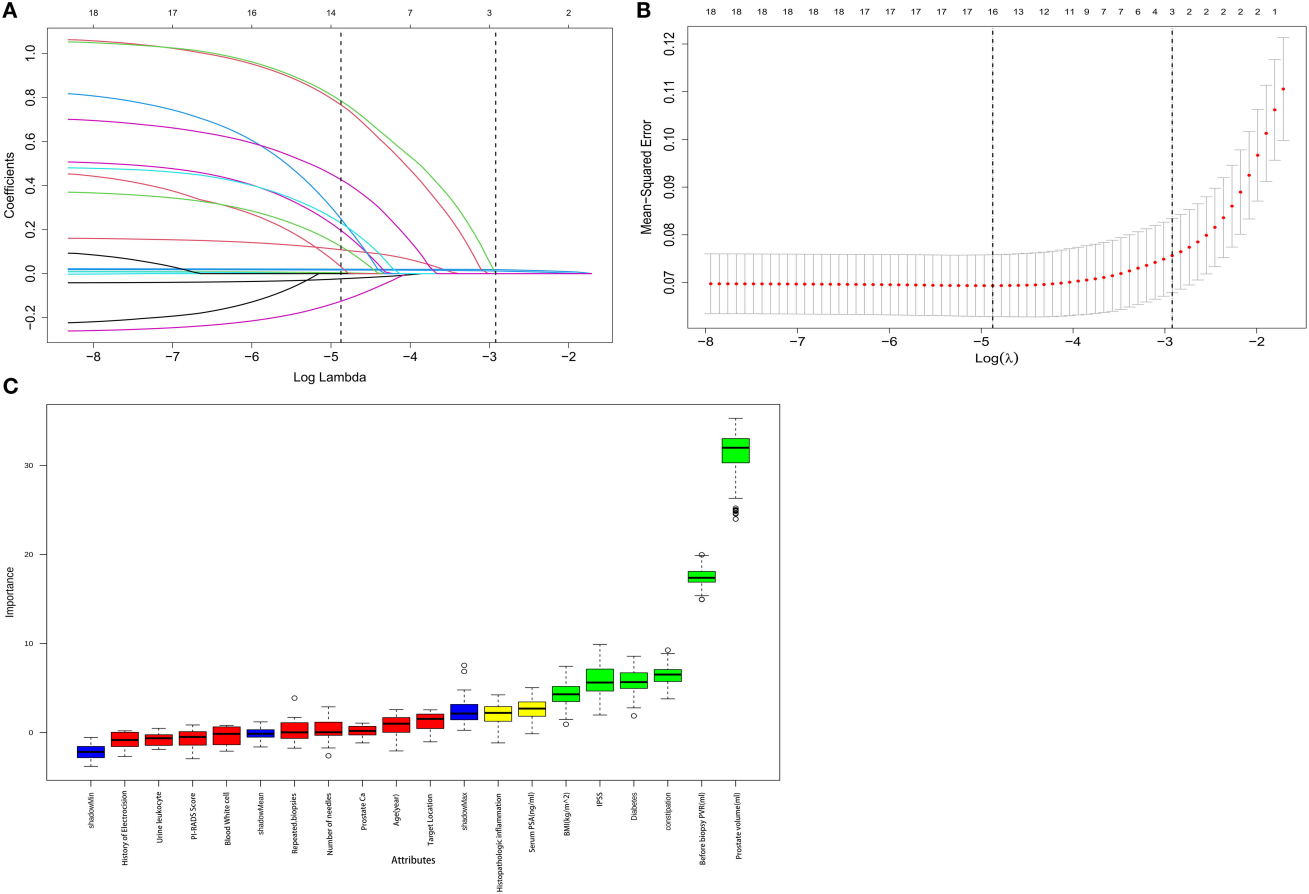
Boruta algorithm is used for feature selection, while LASSO is used for significant variable selection. **(A)** The clinical features’ LASSO coefficient profiles. **(B)** Tenfold cross-validation in LASSO produced the ideal penalization coefficient lambda. The figure displays the minimal mean square error’s lambda value. The most minor absolute shrinkage and selection operator is known as LASSO. **(C)** Boruta’s choice of function. We could counteract the predictive capacity of variables through these randomization features by using blue controls, which were risk permutation characteristics. Green features were validated as applicable; red features did not help predict AUR. The y-axis, linked to the standard deviation derived from 100 iterations, shows the variation in accuracy between each Z-score on the feature and the control. Level thresholds represent significant differences in thresholds between features and controls.

For each univariate logistic regression analysis, we created a forest plot (**Figure 2**). We created a forest plot for each univariate logistic regression analysis (**Figure 2**). We employed LASSO regression to select variables and simplify the model due to the many covariates. We utilized a 10-fold cross-validation strategy for internal validation using Lambda as the dependent variable and pi incidence as the dependent variable. The choice for the λ filter variable was Min. The Lasso method’s numerical variable screening procedure, which uses 14 variable coefficients that fluctuate with penalty coefficients, is shown in **Figure 3A**. The initial integration factor’s coefficient is compressed and eliminated from the model when the coefficient is 0. Every row represents a different variable. The target covariates were ascertained by applying 10-fold cross-validation and the area under the ROC curve (ACU), as illustrated in **Figure 3B**. Two lines represent Lambda. Min and Lambda. Lse and each red dot represent the confidence interval for the appropriate λ value for the covariate of interest. We then used the Boruta feature selection algorithm on optimal parameters to help separate AUR patients from non-AUR patients. Six variables were ultimately chosen, including body mass index, prostate volume, history of diabetes, constipation, IPSS, and residual urine before biopsy (**Figure 3C**). After that, we divided the six variables into groups and used logistic multivariate regression to examine them. The outcomes of the univariate and multivariate logistic regression analyses are shown in **Tables 2** and **3**, respectively.

**Table 2:** Univariate Cox regression analysis of risk factors associated with AUR in patients undergoing TP.

Characteristics	B	SE	OR	CI	Z	P
Age(year)	-0.013	0.01827	0.987	0.987(0.952-1.023)	-0.721	0.471
BMI(kg/m^2)	0.262	0.06779	1.299	1.299(1.141-1.49)	3.858	0
Serum PSA(ng.ml)	0.004	0.00759	1.004	1.004(0.988-1.018)	0.494	0.621
Prostate volume(ml)	0.045	0.00583	1.046	1.046(1.035-1.059)	7.73	0
Urine leukocyte	-0.009	0.01302	0.991	0.991(0.965-1.016)	-0.68	0.497
Blood white cell	-0.01	0.0984	0.99	0.99(0.816-1.201)	-0.102	0.919
History of electrocision	-0.991	0.53748	0.371	0.371(0.109-0.95)	-1.845	0.065
Diabetes	1.644	0.31042	5.178	5.178(2.847-9.673)	5.298	0
constipation	1.872	0.32194	6.504	6.504(3.519-12.52)	5.816	0
Before biopsy PVR(ml)	0.035	0.00464	1.036	1.036(1.027-1.046)	7.591	0
IPSS	0.123	0.01993	1.131	1.131(1.089-1.178)	6.185	0
Repeated biopsies	0.841	0.35673	2.318	2.318(1.119-4.574)	2.357	0.018
PI-RADS Score 4	0.182	0.3591	1.2	1.2(0.598-2.467)	0.508	0.612
PI-RADS Score 5	-0.09	0.38257	0.914	0.914(0.43-1.952)	-0.234	0.815
Target Location	0.94	0.30865	2.56	2.56(1.415-4.777)	3.045	0.002
Number of needles	0.282	0.45824	1.326	1.326(0.579-3.597)	0.616	0.538
Prostate Ca	0.289	0.31827	1.336	1.336(0.727-2.553)	0.909	0.363
Histopathologic inflammation	0.984	0.29808	2.676	2.676(1.492-4.825)	3.302	0.001

AUR, Acute urinary retention; IPSS, International Prostate Symptom Score.

**Table 3:** Multivariate Logistic regression analysis of risk factors for AUR.

Variables	B	SE	OR	CI	Z	P
BMI(kg/m^2)	0.17	0.09227	1.186	1.185 (0.990-1.425)	1.845	0.065
Prostate volume(ml)	0.02	0.00694	1.02	1.020 (1.007-1.035)	2.867	0.004
Diabetes	0.848	0.40028	2.334	2.334 (1.054-5.125)	2.118	0.034
constipation	0.894	0.40652	2.444	2.443 (1.090-5.434)	2.198	0.028
Before biopsy PVR(ml)	0.019	0.00548	1.019	1.019 (1.008-1.030)	3.436	0.001
IPSS	0.006	0.02788	1.006	1.006 (0.951-1.062)	0.23	0.818

AUR, Acute urinary retention; IPSS, International Prostate Symptom Score; PVR, pre-biopsy post-void residual.

### Modal chart model for estimating the risk of AUR following TP

**Figure 4:**
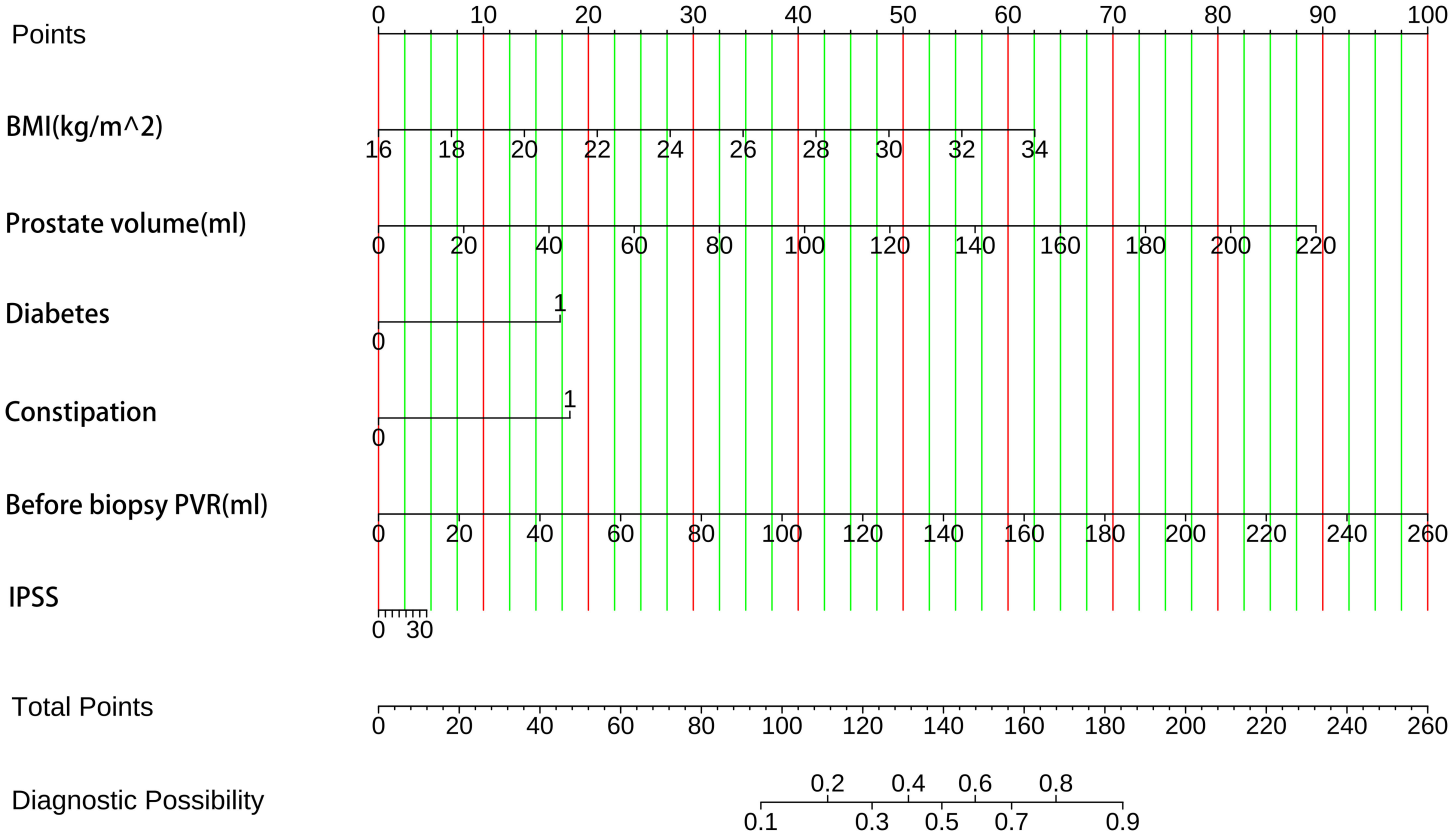
The probability of AUR in TP patients was predicted using clinical nomograms based on multiple logistic regression analysis and Boruta’s algorithm for feature selection. A line was drawn from the matching value to a “dot line” for every indicator to allocate points. By computing the likelihood of similar “totals” and using the individual score totals of the six measurements that comprise the nomogram, we can ascertain the patient’s risk of AUR. BMI, health index. PVR, post-void residual urine volume. IPSS, International Prostate Symptom Score.

The enrolled patients were split into training and test groups in a 7:3 ratio using a randomized stratified grouping technique (**Supplementary Data**). We constructed individualized nomogram estimates to estimate the chance of AUR in patients following TP based on the risk factors determined by binary logistic regression, Boruta feature selection method, and Lasso regression (**Figure 4**). Each element is assigned a score between 0 and 100 on a modal plot, representing its regression coefficient about the requirement of AUR. Adding the scores related to each component may determine the cumulative score, representing an individual’s probability of developing a post-TP AUR. For this calculation, a vertical line from each factor axis intersecting the nomogram’s point axis must be drawn. The total score obtained can then be compared to the total score table for explanatory purposes.

### Validation of nomogram models

**Figure 5:**
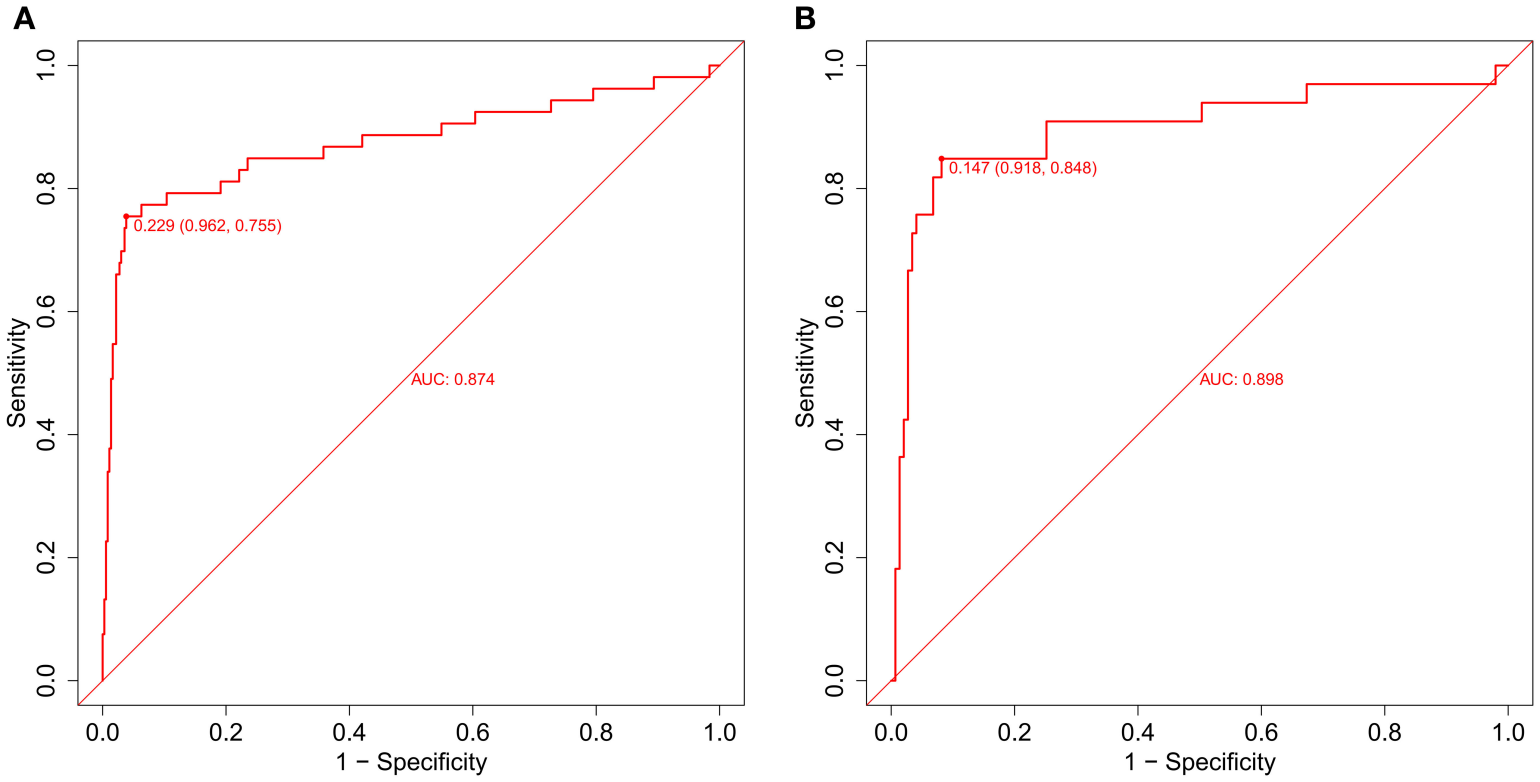
The evaluation and internal validation of the nomogram. **(A)** The AUC of the training group (AUC = 0.874) and **(B)** the validation group (AUC = 0.898) showed that the model had a high discrimination ability.

**Figure 6:**
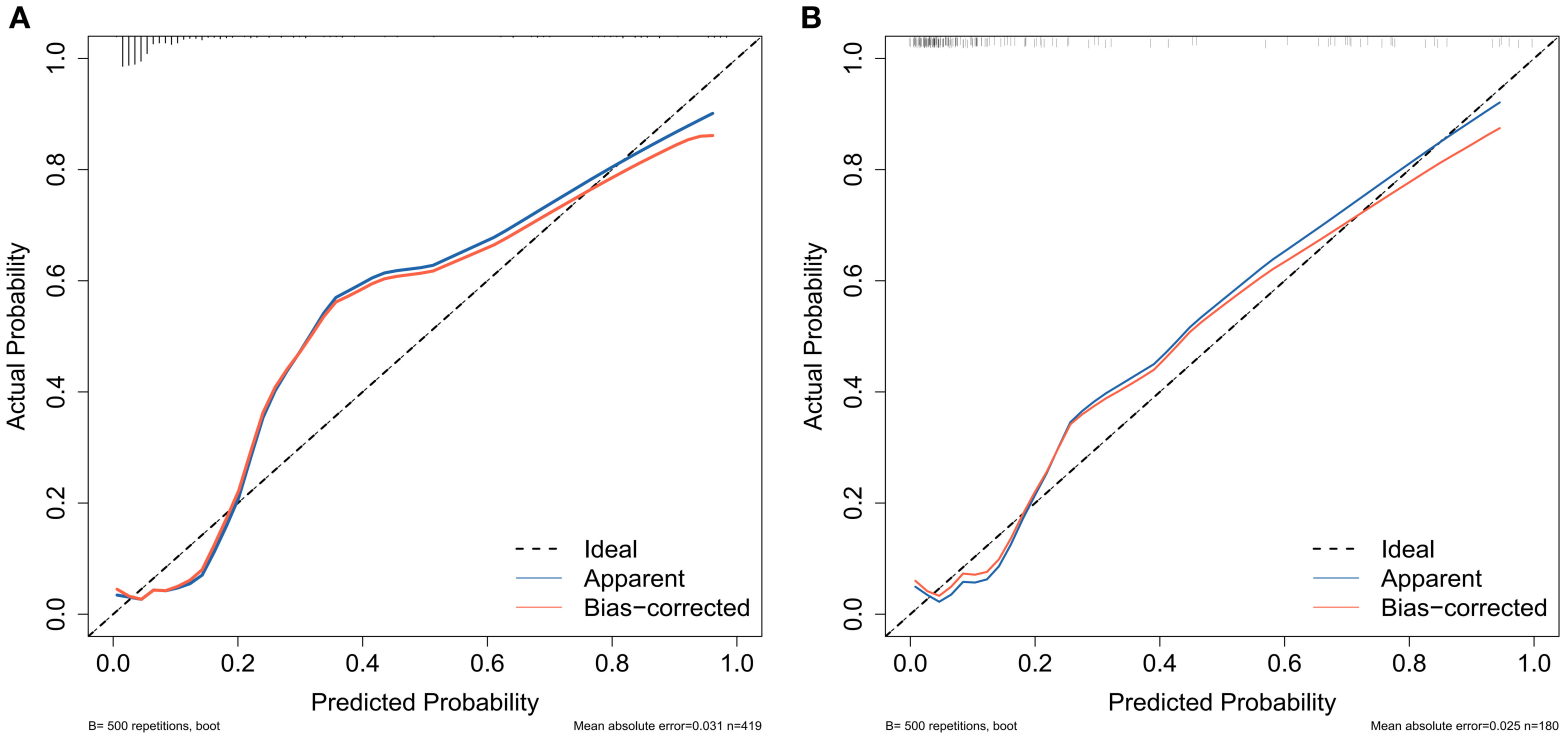
Calibration curves were used to assess the consistency of predicted versus actual risk of AUR following TP. **(A)** calibration curve of the training group and **(B)** calibration curve of the validation group.

For the training cohort model (**Figure 5A**), the AUC was 0.874 (95% CI: 0.806 – 0.9419), and for the testing cohort model (**Figure 5B**), it was 0.898 (0.8218 – 0.9741). The results of the training and testing cohorts showed that the model was effective in discriminating between high and low risk patients. The model was well corrected based on the Hosmer-Lemeshow goodness-of-fit test results for the training cohort (χ2 = 10.797, P = 0.2135) and validation cohort (χ2 = 11568, P = 0.1716). The model’s calibration analysis revealed that the model had been calibrated following 500 internal Bootstrap samplings. For the training set (**Figure 6A**), the Brier score was 0.059 with a p-value of 0.775 (> 0.05), and for the validation set (**Figure 6B**), it was 0.074 with a p-value of 0.697 (> 0.05). The calibration curve revealed a high degree of agreement between the actual likelihood of occurrence and the projected probability.

### Analysis of clinical practicability and rationality of prediction model

**Figure 7:**
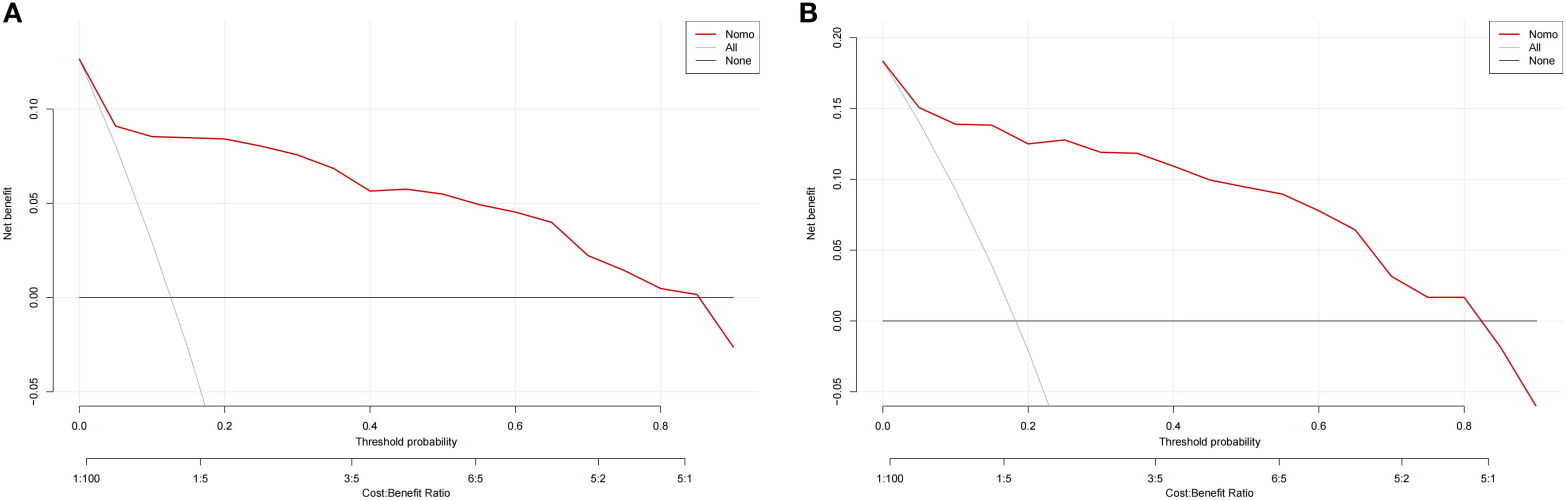
Clinical decision curves for nomogram models. **(A)** denote the decision curve for the training set, and **(B)** denote the decision curve for the validation set.

We employed the incidence of AUR in patients following TP as a state variable and the predicted probability of the calibration plot as a test variable to evaluate the nomogram’s clinical value. As illustrated in **Figure 7**, we created a clinical decision curve (DCA) for the nomogram model. The Y-axis shows net benefit, whereas the X-axis shows threshold probability. A solid gray line denotes that every patient had AUR, while a narrow solid black line shows that none did. The decision curve indicates that the model is clinically useful throughout a comparatively extensive threshold probability range. In contrast, the red curve shows the advantage for patients utilizing the prediction model for this study. Overall, DCA showed a net clinical benefit of the model over a wide range of thresholds, better than single measures (e.g., IPSS, prostate volume).

**Figure 8:**
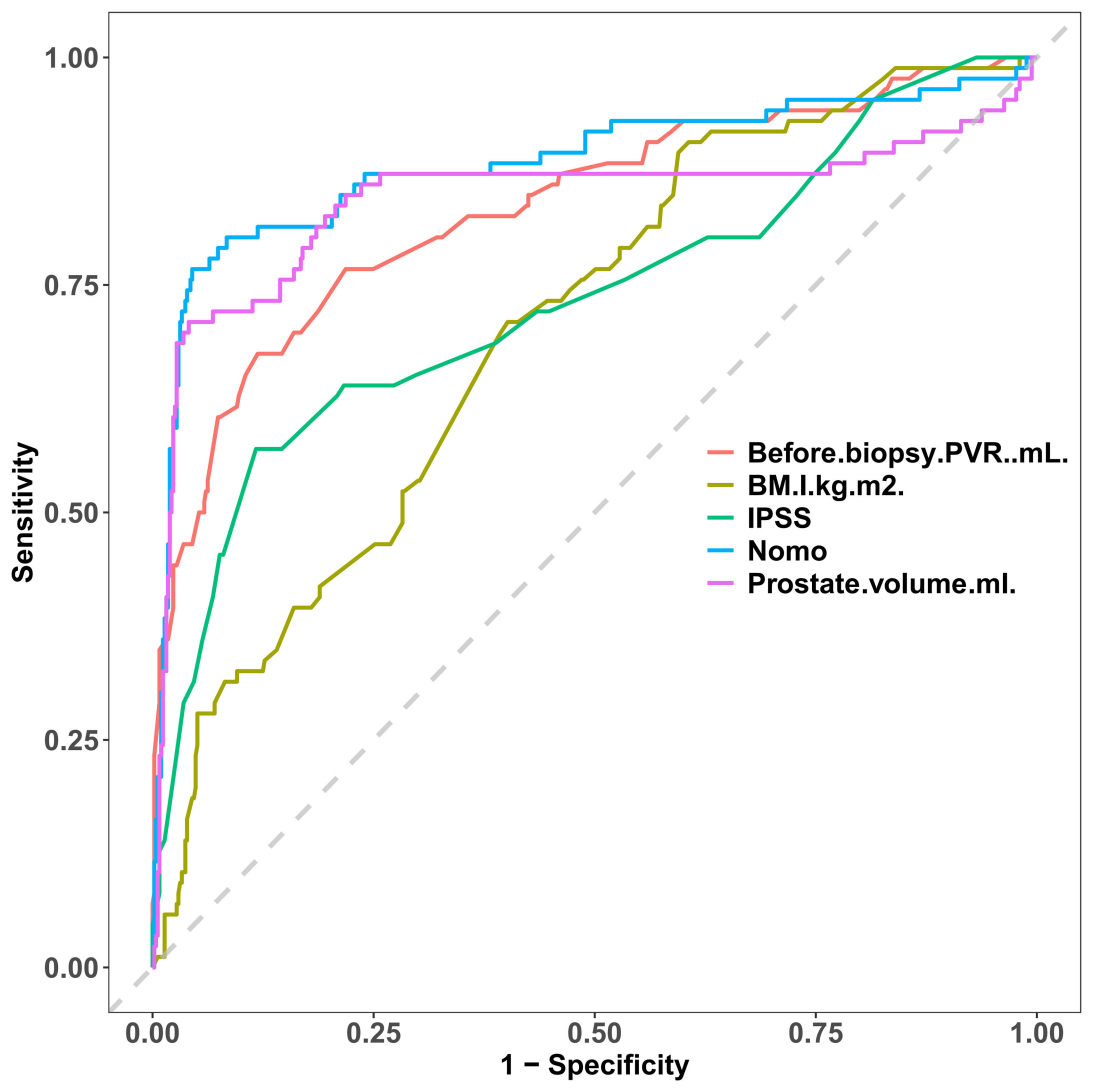
Rationality curve analysis of residual variable risk nomogram.

We contrasted the ROC curves of the predicted nomograms with those of models that only used one predictor to provide a more thorough assessment. In **Figure 8**, the nomogram area under the curve (AUR) was more significant than that of BMI, IPSS, pre-biopsy residual urine, and prostate volume alone, indicating the model’s plausibility; this suggests that the AUR of individual predictors was consistently smaller than that of the predictive model, highlighting the model’s robust performance.

## Discussion

Current studies have mostly focused on the comparison of diagnostic accuracy and complication rates between transperineal and transrectal prostate biopsy in the diagnosis of prostate cancer (27–30). Since template biopsies are carried out more frequently than TRUS biopsies, evaluating any problems and the patient’s following effects is critical. Before template biopsy, it is helpful to understand that postoperative complications, counseling, and patient consent are clinically significant risk factors linked with urine retention. According to reports, between 0.10% and 3% of TP biopsies result in infection (13, 31, 32). AUR occurs in 1.5-13% of patients having TP prostate biopsies (14, 15, 33, 34). Although the frequency of these occurrences has been reported, there is a shortage of information evaluating probable risk factors for infection and AUR following prostate biopsy for TP (13, 14). Predictive models are receiving more attention in several clinical domains, including sepsis, Kawasaki disease, and malignant tumors. Additionally, there is a growing body of pertinent research in these domains (35–38). For AUR following TP, there aren’t many prediction models available yet. Therefore, to support clinical decision-making and offer individualized treatment plans, we set out to create a predictive model for developing AUR following TP surgery.

The clinical information and laboratory parameters of 599 patients who had prostate biopsies were reviewed retrospectively. These included easily accessible laboratory and anthropological data, such as age, sex, BMI, prostate-specific antigen, diabetes, and residual urine before biopsy. We determined the independent variables for AUR in TP patients to be body mass index, prostate volume, history of diabetes, constipation, IPSS, and residual urine before biopsy using various statistical techniques. We created a straightforward and precise nomogram, verified it within the model, and demonstrated its clinically solid applicability and efficacy.

In 14.3% of our cohort, urinary retention happened. Urinary retention has an incidence ranging from 1.6 to 11.4%, according to the NICE guidelines for transperineal template biopsy of the prostate (12, 33). Pepe et al. (34) conducted a large single-center study involving 3000 patients with varied amounts of biopsies, and they found a low mean incidence of urine retention of 6.7%. Our rate was compared to other groups (39), even though it was marginally higher than the incidence reported by NICE, as reported by Merrick et al. Urinary retention occurs 1.7% of the time in patients receiving TRUS biopsy, according to a comprehensive review by Loeb et al. (11). In contrast to transperineal biopsy, which typically requires at least 24 cores, the conventional protocol for TRUS biopsy only requires 12 cores. Urinary retention can also be prevented by reducing the number of biopsies performed. Taking targeted biopsies instead of entire templates can also save operating time. However, it is contingent upon the operator’s cognitive objectives. We selected targeted + systematic access, a total of 16 needles, for patients with apparent targets on MRIs, which may be why the number of biopsy needles we used did not lower the rate of urine retention.

In line with our findings, several studies have found a positive correlation between the frequency of urine retention and the number of needle biopsies performed; however, this relationship was not statistically significant (33, 34). Urinary retention was linked to a prostate volume of more than 68 milliliters, according to Willis et al. (22). Increased prostate volume has also been found in several studies to be an independent predictor of AUR diagnosis after TP (7, 14). The ratio of the transitional zone volume to the total prostate volume and a greater IPSS are two additional prostate volume markers that may predispose patients to urine retention and worsen LUTS (11). Urinary retention was significantly predisposed to the severity of LUTS in our study. Furthermore, it has been suggested that more enormous prostate volumes and higher baseline IPSS values may be indicators of LUTS and AUR following PB (40). Consequently, discussing risk with patients could be considered a preoperative risk factor. Constipation can be regarded as a significant and trustworthy predictor of AUR in individuals receiving transrectal ultrasonography-guided prostate biopsy, according to research by Cahit Sahin et al. (41). Acute postoperative urine retention has also been described in our community and is also connected with diabetes mellitus and aging (11). One known consequence of diabetes is neuroautonomic dysfunction, which has been linked to an increased risk of urine retention. According to other research, diagnosing chronic urine retention (CUR) is typically more challenging. It is generally related to higher levels of postvoid residual urine (PVR) (42), and our data imply that PVR before biopsy is a separate risk factor for AUR following TP. Ultimately, our research revealed that AUR patients’ BMIs were greater than non-AUR patients. This appears to be a discovery. No study has been done on how BMI affects AUR following TP biopsy. BMI is a little-studied topic, and while we show some intriguing findings, further research is needed to draw firm conclusions.

Existing studies mostly focus on complications after transrectal puncture (TR), there are very few studies on AUR after TP, and most of them are univariate analysis (such as only focusing on prostate volume or number of puncture needles); a few studies involving TP do not establish prediction models and do not include machine learning algorithms to optimize variable screening (29, 43, 44). As Francesca Kum et al. found factors for urinary retention after transperineal template biopsy of the prostate, but only a univariate association, no predictive model was constructed (14). Sabri Cavkaytar et al. (45) used logistic regression to analyze risk factors for postpartum urinary retention, but given the high dimensionality of the data, no further dimensionality reduction was performed using machine learning algorithms. Samuel L Malnik et al. used machine learning to develop a postoperative predictive model for urinary retention after lumbar spine surgery, but only LASSO regression models were used, but often this was not able to eliminate random fluctuations in factor interactions (46). Moreover, the AUC of our two machine learning algorithms was more significant than that of Ding Xuexuan et al., who identified the core genes of asthma using five machine learning algorithms (21).

The clinical nomogram of this study integrates biomarkers and clinical characteristics, includes factors such as constipation and BMI in the prediction model of AUR after TP for the first time, and improves the rigor of variable screening through a two-feature selection algorithm to provide a personalized assessment of whether to continue Foley catheter or take additional safety measures to prevent AUR in TP patients. The nomogram constructed in this study is based on six easily accessible clinical measures (body mass index, prostate volume, history of diabetes, constipation, International Prostate Symptom Score, preoperative residual urine volume) that can be directly integrated into the preoperative assessment process of transperineal prostate biopsy (TP). Clinicians achieve precise stratified management by rapidly calculating the patient ‘s acute urinary retention (AUR) risk score: for example, high-risk patients (score > 70%): prophylactic use of alpha-blockers to relax the bladder neck preoperatively, or prolonged monitoring to 72 hours postoperatively, timely detection of signs of urinary retention, and reduction in the risk of complications such as emergency catheterization rate and overdistension of the bladder; for low-risk patients (score < 30%): use a standardized discharge regimen to reduce unnecessary inpatient observation and reduce medical costs while ensuring safety.

The current study has some limitations. It is a single-center cross-sectional study with a limited sample size that may introduce selection bias. In addition, we only performed internal validation on nomogram models, and subsequent studies also required external validation. Furthermore, potential influencing factors such as the number of puncture needles and operator experience were not included, and subsequent studies could expand the range of variables. Subsequent studies can be conducted in a large sample, multicenter, prospective study to find more risk factors for AUR complications in TP patients so that relevant measures can be taken early to avoid repeated catheterization and further hospitalization and improve patient discomfort.

## Data Availability

The original contributions presented in the study are included in the article/**Supplementary Material**. Further inquiries can be directed to the corresponding authors.
